# A Matched Cohort Study of Standard Chemo-Radiotherapy versus Radiotherapy Alone in Elderly Nasopharyngeal Carcinoma Patients

**DOI:** 10.1371/journal.pone.0119593

**Published:** 2015-03-13

**Authors:** Qi Zeng, Yan-Qun Xiang, Pei-Hong Wu, Xing Lv, Chao-Nan Qian, Xiang Guo

**Affiliations:** 1 State Key Laboratory of Oncology in South China, Collaborative Innovation Center for Cancer Medicine, Guangzhou, China; 2 Department of Medical Imaging & Interventional Radiology, Sun Yat-sen University Cancer Center, Guangzhou, China; 3 Department of Nasopharyngeal Carcinoma, Sun Yat-sen University Cancer Center, Guangzhou, China; University of Science and Technology of China, CHINA

## Abstract

The impact of standard chemo-radiotherapy (CRT) as preferred therapy for elderly patients (age≥60 years) with nasopharyngeal carcinoma (NPC) remains unclear. Therefore, a strict matched cohort study was conducted to compare the survival and treatment toxicity of standard chemo-radiotherapy in the elderly NPC patients with those of radiotherapy (RT) alone. From 1998 to 2003, total 498 newly diagnosed elderly non-metastatic NPC patients were abstracted and classified into two groups by the treatments they received. For each patient in the CRT group, a matched pair in RT group was identified by matching for gender, age, histological type, T and N classifications, RT dose to primary tumor and neck nodes, and days of radiotherapy. Treatment tolerability and toxicity were clarified, and treatment outcomes were calculated and compared between the two groups. Two groups were well balanced in clinical characteristics because of the strict matching conditions. Totally 87 pairs can be assessed according to the criteria. The 5-year OS, CSS, FFS, and LR-FFS for CRT and RT groups were 62% versus 40% (*P*=0.013), 67% versus 47% (*P*=0.018), 65% versus 53% (log-rank: *P*=0.064, Breslow: *P*=0.048), and 88% versus 72%, (*P*=0.019), respectively. There was no significant difference in 5-year D-FFS between the two groups (75% vs. 73%, *P*=0.456). The CRT group experienced significantly more Grade ≥3 acute mucositis (46.0% vs. 28.7%, *P*= 0.019). We concluded that standard chemo-radiotherapy can achieve a reasonable local and regional control in elderly NPC patients with acceptable and reversible acute toxicity. However, distant metastasis remains the dominant failure pattern. When the elderly NPC patients are in good performance status following a complete evaluation of overall functional status and comorbidity conditions, standard chemo-radiotherapy is worthy of recommendation.

## INTRODUCTION

The frequency of nasopharyngeal carcinoma (NPC) varies extensively with age, ethnic and geographical origin [1–2]. The highest NPC incidence occurs in Southern China, with the yearly incidence varying between 15–50/10^5^ population, followed by Singapore and among individuals of Chinese descent in the United States [3]. Most NPCs in endemic areas commonly arise between the fourth to sixth decades of life and subsequently decline, but their occurrence in the elderly population is not rare [4].

Three previous meta-analyses and several other prospective randomized trials that were conducted in endemic areas have demonstrated that the addition of chemotherapy to radiotherapy could significantly improve survival in NPC patients [5–9]. Therefore to date combined chemo-radiotherapy has become the standard regimen for NPC patients. However elderly NPC patients accounted for only a small part of these studies because of the strict inclusion criteria, optimal treatment strategy for elderly NPC patients remains unclear.

When we are faced with elderly NPC patients, we would have to consider two questions: First, whether elderly patients can receive aggressive combined treatment? Second, whether chemotherapy is able to improve the survival of elderly patients? For the first question, an effective assessment is essential to establish the benefits and risks of anti-neoplastic treatments in the elderly population. We can select patients with limited disease and good performance status (PS or KPS score). A more multidimensional, reliable evaluation has been provided by the Comprehensive Geriatric Assessment (CGA) [10]. As for the second question, there were few publications in the literature concerning elderly NPC, and this part of the population was often excluded from the randomized clinical trials [11]. Previous studies about elderly NPC from our department have been published [12–13], but there were still some imperfect aspects, such as the matched conditions did not include some established prognostic factors. Moreover, maybe it couldn’t reflect the greatest number of elderly NPC to select 65-year-old as the cutoff point of elderly NPC according to epidemiology of NPC from endemic regions of China.

Thus, in the present study, we conducted a more strict matched cohort analysis of survival and treatment toxicity in elderly NPC patients (age≥60 years) received standard CRT versus RT alone.

## MATERIALS AND METHODS

The patients selected in the present study were hospitalized between January 1998 and December 2003 in the Department of Nasopharyngeal Carcinoma, Sun Yat-sen University Cancer Center. The study was approved by Hospital Ethics Committee and the institutional reviewed board at Sun Yat-sen University Cancer Center, Guangzhou, China. This was a retrospective analysis of routine data and therefore we requested and were granted a waiver of individual informed consent from the ethics committee. Patients were eligible for this study if they met the following inclusion criteria: (i) Elderly patient who is 60 years or older; (ii) Histologically confirmed nonkeratinizing or undifferentiated NPC (World Health Organization type II or III); (iii) KPS (Karnofsky performance scale) score ≥80; (iv) Without evidence of systemic metastasis; (v) Completion of the scheduled total radiotherapy dose.

**Fig 1:**
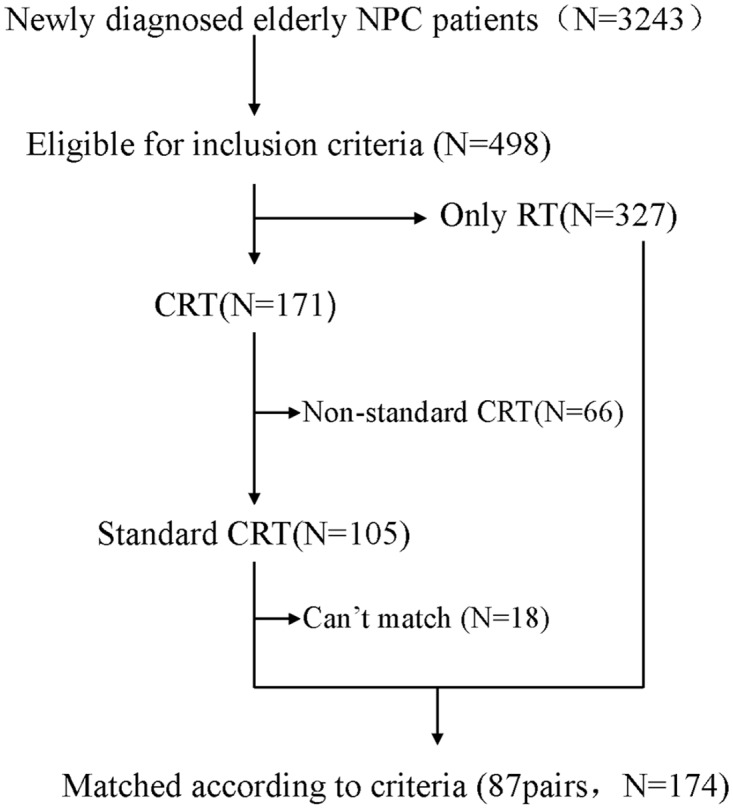
Flowchart of patients. Footnote: NPC, Nasopharyngeal carcinoma; RT, Radiotherapy; CRT, Chemoradiotherapy; CT, Chemotherapy.

Fig. 1 showed the flowchart of patients. Totally 498 eligible cases of elderly NPC patients were included in this study. The elderly patients were classified into two groups by the treatments they received, basically with or without chemotherapy. For each patient in the CRT group, a matched pair in RT group (the reference group) was identified by matching for gender, age (the difference of two group, ≤ 3 years), histological type, T and N classifications (Chinese 1992 staging system)[14–15], radiotherapy dose to primary tumor (the difference of two groups, ≤ 2Gy) and neck nodes (the difference of two groups, ≤ 2Gy), and days of radiotherapy (the difference of two groups, ≤ 5 days). For the cases a matched pair could not be appropriately identified were dropped from the subsequent analysis. Whenever multiple matches were possible, the RT patient whose treatment date was closest to the CRT group patient was selected. Data were manually cross-checked after export to SPSS, with random-sampling verification of the matching process.

### Radiotherapy

All patients were treated by curative-intent RT. Details of our RT technique in Sun Yat-Sen University Cancer Center have been reported previously [15]. Basically, the patients were treated with two lateral-opposing faciocervical portals to irradiate the nasopharynx and the upper neck in one volume followed by the shrinking-field technique (two lateral opposed facial fields) to limit irradiation of the spinal cord. An anterior cervical field was used to treat the neck with a spinal cord block. The accumulated radiation doses were 64–72 Gy to the primary tumor, 60–66 Gy to the involved areas of the neck, and 48–50 Gy to the uninvolved areas. The median overall radiotherapy time of each group was 52 days (range: 49–70) without inclusion of time for boost.

Booster portal was done if necessary. Different radiation energies, including megavoltage photons (cobalt-60, 6 MV or 8 MV) and electrons were used. In case of nasal or ethmoidal involvement, an anterior facial electron field was added. Patients with bulky parapharyngeal disease were boosted with the “parapharyngeal boost technique” as described by Tsao[16]. A booster dose (8 to 10 Gy per four to five fractions) was delivered to the skull base in patients with involvement of the skull base.

### Chemotherapy

Totally 87 patients received standard chemo-radiotherapy which was defined as at least 2 cycles of induce chemotherapy or weekly cisplatin concurrent chemotherapy for at least five cycles or 3-weekly concurrent regimens for three cycles. Two cycles of induction chemotherapy was administrated in 56 out of 87 patients in the CRT group. The induction chemotherapy was a combination of cisplatin and 5-FU, with cisplatin (30 mg intravenously) given on Days 1–5 and 5-fluorouracil (750 mg intravenously) on Days 1–5, repeated every 21 days. An analysis of chemotherapy delivery showed patients often received fixed lower total doses of each drug regardless of body surface area, primarily as a result of dose reduction, rather than dose adjustment.

Weekly concurrent chemotherapy with cisplatin single drug regimen was given in 19 out of 87 patients, with cisplatin (30–40mg/m^2^ on Day 1) given intravenously weekly for 5–7 weeks concurrently with the RT. While 3-weekly concurrent regimens was given in 7 out of 87 patients, with cisplatin (80–100mg/m^2^) given intravenously 3-weekly for three cycles.

In addition, 5 patients received 2 cycles of induction chemotherapy and thereafter 1–2 cycles of cisplatin concurrent chemotherapy.

### Patient assessments and following-up

#### Short-term efficacy

The short-term treatment efficacy was evaluated in the first 3 months after completion of the therapy. The work-up for this evaluation included physical examination, mirror examination of the nasopharynx or nasopharyngeal endoscopy, MRI or CT scanning of the nasopharynx and neck, chest X-ray, and abdominal ultrasonography. Tumor extinction criteria included complete disappearance of the loco-regional lesions in physical and endoscopical examination, and tumor regression in MRI or CT scanning with the basic structure of the paranasopharyngeal soft tissues back to normal. The toxicities were graded using the Common Terminology Criteria for Adverse Events (CTCAE) v4.0[17]. RT-related toxicities were graded according to the Radiation Morbidity Scoring Criteria of the Radiation Therapy Oncology Group [18]

#### Following-up

The follow-up duration was calculated from the day of beginning therapy to either the day of death or the day of the last examination. For all of the patients, 98.3%, 97.1% and 91.3% had complete following-up at l, 3, 5 year, respectively. The median follow-up for the whole group was 52 months (range: 1.4–118 months), for alive patients was 64 months (range:3–118 months).

### Statistical analysis

The goal of the present study was to assess and compare overall survival rates (OS), cancer-specific survival rates (CSS), failure-free survival rates (FFS), locoregional failure-free survival rates (LR-FFS), distant failure-free survival rates (D-FFS), and treatment related toxicities between RT and CRT groups. OS was defined as the length of time between the date of beginning therapy and death from any cause. CSS was defined as the length of time between the date of beginning therapy and death from NPC. FFS was defined as the length of time between the date of beginning therapy and the date of treatment failure or death from any cause, whichever was first. For LR-FFS and D-FFS analyses, the latencies to the first locoregional or remote failure, respectively, were recorded. For LR-FFS analysis, the first locoregional failure was scored. Patients who died of distant metastases or intercurrent illnesses without locoregional recurrence were censored. A similar definition was used for D-FFS analysis. SPSS 16.0 statistical software was used. The Life tables and Kaplan-Meier method were used for calculation of OS, CSS, FFS, LR-FFS, and D-FFS. Toxicity rates were compared using the chi-square test (or Fisher’s exact test, if indicated). The statistical significance of differences among survival curves was analyzed using the log-rank test or Breslow test. A two-tailed p value of less than 0.05 was considered statistically significant.

## RESULTS

### Characteristics of elderly NPC patients

From January 1998 to December 2003, 3243 newly diagnosed NPC patients were treated in our department. Among them, 498 eligible cases of elderly NPC patients were selected for the subsequent study, which composed of 15.36% (498/3243) of all the NPC patients. According to their treatment, 171 (34.5%) patients underwent combined treatments and 327 (65.5%) patients underwent RT alone, which was selected as the reference group. Among the 171 patients, 105 cases completed the scheduled cycles of chemotherapy which was defined as standard chemo-radiotherapy previously. All of the 105 cases were appropriately matched according to criteria to the reference group. A total of 87 pairs could be assessed according to the matching criteria (Fig. 1). The two groups were well balanced because of accordance with the strictly matching conditions. The ratio of male to female in each group was 5.21:1, with 73 males and 14 females. The median age was 64 (range: 60–70) in CRT group, 65 (range: 60–73) in RT group. In each group, proportions of T stage diseases (T1:T2:T3:T4 = 4:16:35:32), N stage diseases (N0:N1:N2:N3 = 19:25:33:10) and clinical stages (III: IV = 43:44) were balanced.

### Clinical response

The complete response at the primary site was 90.8% for patients that received RT alone versus 94.3% for CRT patients at the end of treatment. Three months after RT, complete response in RT group versus CRT group was 100% versus 98.9%. Similarly, the complete response at the neck region was 82.4% for RT patients versus 83.8% for CRT patients at the end of treatment, while 3 months after RT, it was 98.5% versus 97.1%; When both the primary site and the neck were considered together, the composite overall complete response rates were 99.4% for RT alone versus 98.3% for CRT (*P* = 0.315).

### Survival

**Fig 2:**
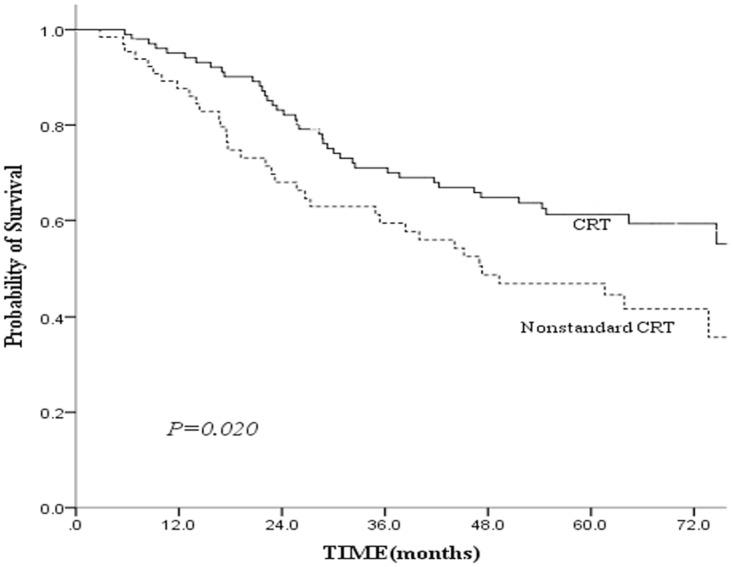
Comparison of cancer-specific survival between CRT and nonstandard CRT groups. Footnote: CRT, standard chemo-radiotherapy; Nonstandard CRT, nonstandard chemo-radiotherapy.

To clarify the impact of completion of the scheduled chemotherapy or not on the survival of elderly NPC patients, we compared CSS rates between 105 patients received standard CRT and 66 patients received nonstandard CRT before matched-pair analysis. The 5-year CSS rates of the above two groups were 56% and 36%, respectively (Fig. 2, *P* = 0.020). In order to eliminate the potential bias from nonstandard CRT group, patients completed the standard CRT were selected for the subsequent matched-pair analysis.

**Fig 3:**
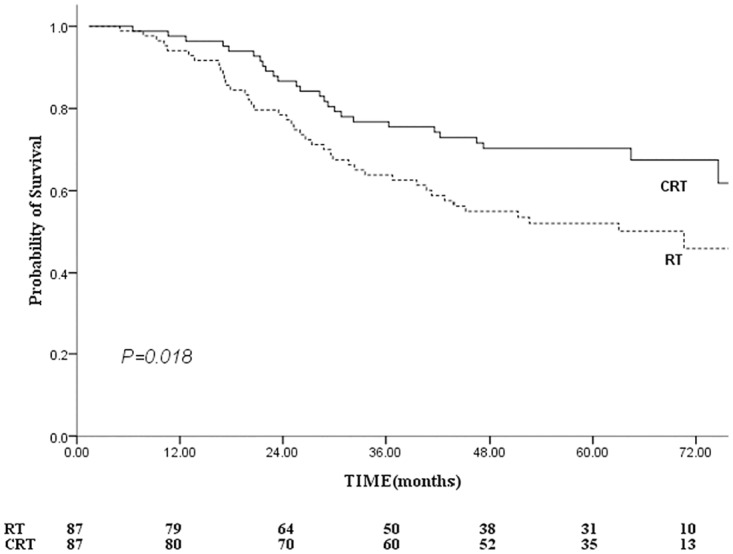
Comparison of cancer-specific survival between the combined chemo-radiotherapy and radiotherapy alone groups. Footnote: RT, radiotherapy; CRT, standard chemo-radiotherapy.

For the 87 pairs elderly NPC patients, 81 deaths (49 in the RT group and 32 in the CRT group) were reported, of which 67 (41 in the RT group and 26 in the CRT group) were related with NPC. Other causes of death in the RT group included cerebrovascular accidents and injury. Other causes of death in the CRT group included heart failure, pneumonia and senescence. The median CSS time was 72 versus 64 months in the CRT versus RT groups, respectively. The 5-year OS rate for the whole group was 50%. A 5-year OS rate of 62% was observed in CRT group compared with 40% in RT group (*P* = 0.013). The 5-year CSS rate in the CRT group was 67% compared with 47% in the RT group (Fig. 3, *P* = 0.018).

### Patterns of treatment failure

The patterns of treatment failure were summarized in Table 1. Distant metastasis was a more important treatment failure factor than locoregional failure in the CRT group (20.7% total distant failure versus 10.3% total locoregional failure). This difference was of marginal statistical significance (chi-square = 3.551, *P* = 0.06); For RT group, distant metastasis and locoregional failure have similar ratios (23.0% vs. 24.1%).

**Table 1:** Incidence and site of progression or recurrent diseases.

Site	RT group (n = 87)		CRT group (n = 87)	
	No.	%	No.	%
Local only	11		5	
Local and nodal	2		1	
Local and distant	2		0	
Local, nodal and distant	0		0	
Nodal only	5		3	
Nodal and distant	1		0	
Distant only	17		18	
Total locoregional failure	21	24.1	9	10.3
Total distant failure	20	23.0	18	20.7
Total	38	43.7	27	31.0

Footnote: RT, radiotherapy; CRT, standard chemo-radiotherapy.

**Fig 4:**
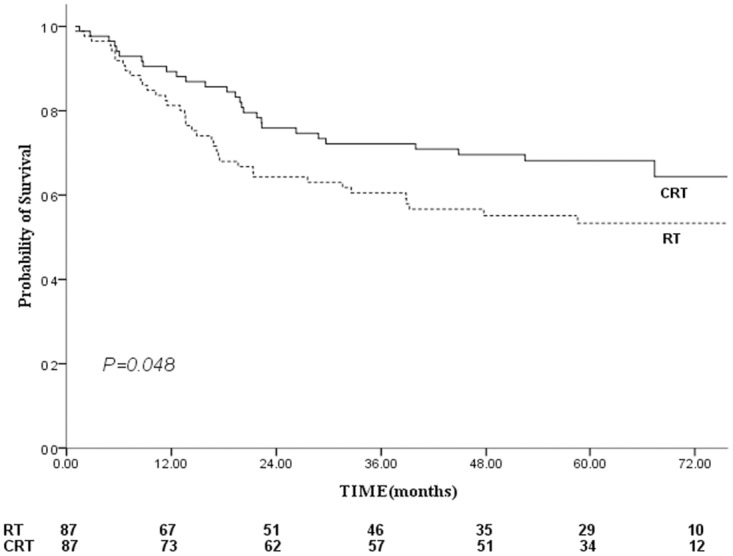
Comparison of failure-free survival between the combined chemo-radiotherapy and radiotherapy groups. Footnote: RT, radiotherapy; CRT, standard chemo-radiotherapy.

**Fig 5:**
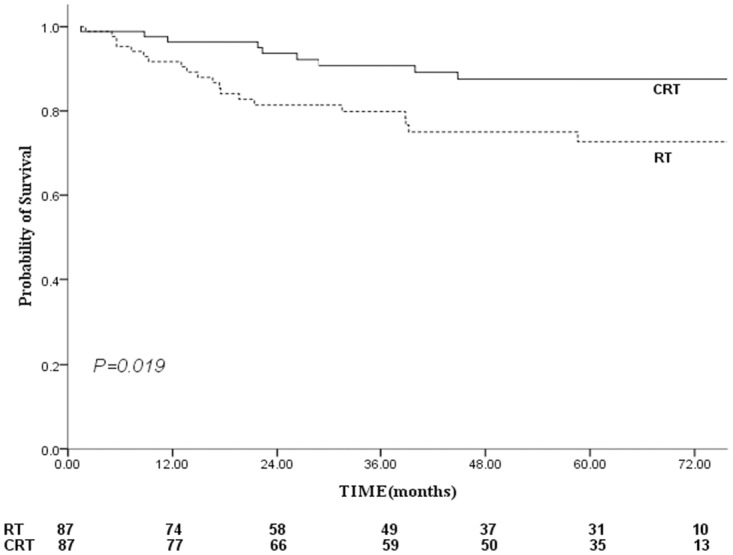
Comparison of locoregional failure-free survival between the combined chemo-radiotherapy and radiotherapy groups. Footnote: RT, radiotherapy; CRT, standard chemo-radiotherapy.

The 5-year FFS were 65% versus 53% for the CRT versus RT groups, respectively (Fig. 4), this difference was marginal statistical significant (log-rank: *P* = 0.064, Breslow: *P* = 0.048). The 5-year LR-FFS for the CRT group were greater than that for the RT group (88% vs. 72%, *P* = 0.019; Fig. 5). Therefore, patients who receive CRT had a reduced risk of local-regional relapse, with a hazard ratio of 0.403 (95% CI, 0.183–0.886; *P* = 0.024).

**Fig 6:**
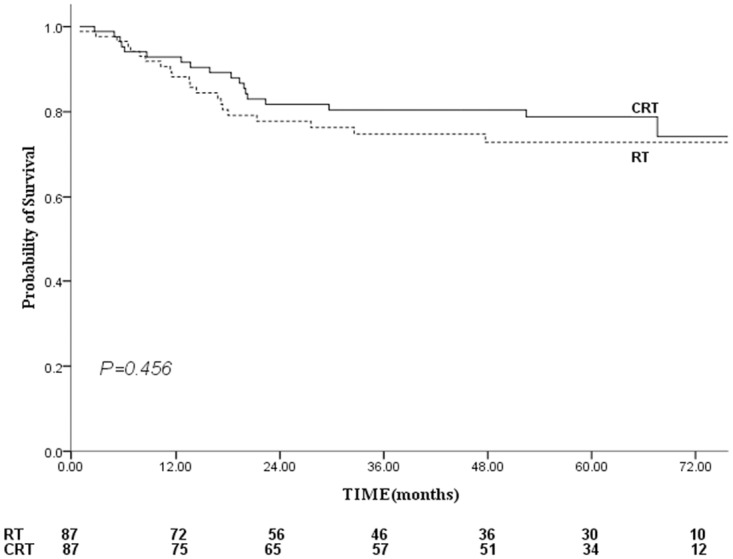
Comparison of distant failure-free survival between the combined chemo-radiotherapy and radiotherapy groups. Footnote: RT, radiotherapy; CRT, standard chemo-radiotherapy.

As shown in Fig. 6, The 5-year D-FFS for the CRT and RT groups were 75% and 73% (*P* = 0.456), respectively. Patients were considered high distant metastases risk if they had N2–3 cervical lymph node disease. A subgroup analysis was performed to examine the impact of chemotherapy on patients with N2–3 disease. The 5-year D-FFS in the CRT and RT groups with N2–3 disease were 76% and 74%, respectively (*P* = 0.435). Chemotherapy could not prevent distant metastases in patients with N2–3 disease.

### Treatment toxicity

CRT was associated with a notably higher incidence rate of toxicity than RT alone. For hematologic toxicity, the incidence of leukopenia (81.6% vs. 24.1%, *P* = 0.000), neutrocytopenia (18.4% vs. 0%, *P* = 0.000), platelets abnormal (17.2% vs. 1.1%, *P* = 0.000), and anemia (48.3% vs. 13.8%, *P* = 0.000) were significantly higher in the CRT group. The incidence of Grade ≥3 toxicities was also higher in the CRT group, which occurred in 8.0% of patients for leucopaenia, 6.9% of patients for anaemia. With respect to nonhematologic toxicity, the incidence of Grade ≥3 acute mucositis (46.0% vs. 28.7%, *P* = 0.019), and Grade ≥2 weight loss (35.6%vs. 18.4%, *P* = 0.01) were significantly higher in the CRT group. There was no obvious liver or renal function impairment. Details of toxicity types were listed in Table 2.

**Table 2:** Patients experiencing the toxicity (n = 174).

Toxicity	RT group (toxicity grade)				CRT group (toxicity grade)				*p*
	1	2	3	4	1	2	3	4	
Hematologic									
Leukopenia	14	6	1	0	29	35	7	0	0.000
Neutrocytopenia	0	0	0	0	5	4	6	1	0.000
Thrombocytopaenia	1	0	0	0	8	6	0	1	0.000
Anemia	6	4	2	0	17	19	3	3	0.000
Nonhematologic									
Mucositis	34	28	25	0	18	29	40	0	0.019
Weight loss	33	16	0		30	30	1		0.010

Footnote: RT, radiotherapy; CRT, standard chemo-radiotherapy.

## DISCUSSION

**Fig 7:**
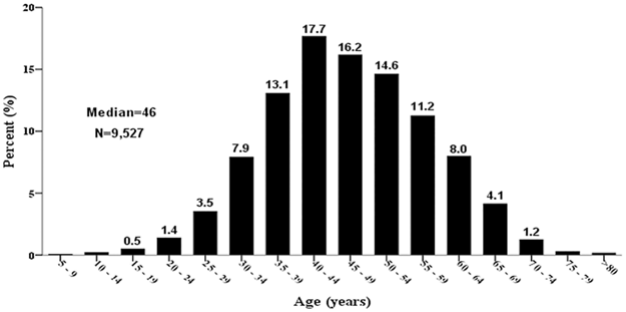
Distribution of patients with nasopharyngeal carcinoma according to their age (database from our department, 1992–2003).

Little has been published regarding the definition therapy of elderly NPC patients. In previous elderly NPC studies, oncologists selected 60, 65 or 70 years as the cutoff point of elderly NPC [12–13,19–20]. Fig. 7 shows the distribution of patients with NPC grouped by age in our institution. We have selected 60-year-old as the cutoff point which can reflect the greatest number of elderly NPC according to epidemiology of NPC from endemic regions of China because more than 80% of the patients with NPC were aged between 30 and 59 years. Although NPC incidence remains steady or increased slightly in Southeastern China [21], the number of elderly NPC patients is increasing with the rapidly rising proportion of elderly persons, and it is therefore essential to examine optimal treatment for the elderly in the near future.

For a long time elderly patients have not been considered good candidates to receive aggressive therapy. Complications of cytotoxic chemotherapy are more common in older cancer patients than in younger one. A decline in organ function can alter the pharmacokinetics of many commonly used chemotherapeutic agents in some elderly patients, making toxicity less predictable [22–23]. Comorbidity, which increases with age, is thought to play an important role in oncologists’ motives to treat the elderly less intensively [19,24]. As a result, elderly patients have been underrepresented in clinical trials, and patients often undergo less combined treatments secondary to age-related biases [25]. As a study of head and neck cancer patients has shown, even the healthy elderly were less likely to receive standard treatment than younger patients [26]. Previous study showed only 38.1% (306/804) elderly patients (age≥65 years) with advanced NPC received combined chemo-radiotherapy [12], which is similar as our report. Although there were not more comorbidities, only 34.5% (171/498) patients underwent combined treatments. In those patients, 105 cases received the cycles of chemotherapy sufficiently which we called standard chemo-radiotherapy (at least 2 cycles of induce chemotherapy or weekly cisplatin concurrent chemotherapy for at least five cycles or 3-weekly concurrent regimens for three cycles). Furthermore, various data show elderly patients with appropriate patient selection derive a benefit from standard chemotherapy regimens that is equal to that of younger patients [27–28]. Therefore patients who received standard chemo-radiotherapy were chosen arbitrarily as subjects. A matched cohort analysis was employed, using all established prognostic factors for survival, locoregional control, and distant metastasis, to eliminate the potential bias. Although this analysis does not have the value of a prospective randomized trial, it provides a balanced evaluation of the impact of chemotherapy on the outcome of elderly NPC patients.

NPC is a highly chemosensitive tumor [29–30]. The effective of chemotherapy is related to dose-dense, but our data showed that elderly NPC patients often received fixed lower total doses of each drug regardless of body surface area, primarily as a result of dose reduction, rather than dose adjustment, which can also be seen in the other study [20]. In real clinical practice, on the condition of no guidelines in elderly NPC patients, many oncologists attached importance to the toxicities caused by CRT and preferred a lower dose without evaluation. This conservative treatment selection may prevent some elderly patients from longer survival [12].

CSS is used in the present study in order to exclude death due to comorbidities. A previous study showed favorable 2-year survival in elderly NPC patients, with OS, DFS, LRRFS and DMFS observed at 87%, 73%, 92% and 76%, respectively, but their sample was relatively small (26 patients were analyzed) [20]. The results of this study showed a clear survival advantage after the addition of chemotherapy to the treatment, and improvements in OS, CCS and LR-FFS were all observed in the CRT group (Figs. 3, 5). The median CSS time was 72 and 64 months for the CRT and RT groups, respectively. The 5-year CSS rates were 67% for the CRT group and 47% for the RT group (*P* = 0.018). This gain mainly due to the incidence of locoregional failure was significantly lower in CRT group and the improvement in LR-FFS (88% vs. 72%, P = 0.019) could have translated into a CSS benefit. It can be explained that the addition of chemotherapy to shrink the tumor, leading to an increased safety margin between the tumor volume and the radiation volume [15,31]. Then tumor-cell clonogens could be sensitized by the delivery of chemotherapy and ionizing radiation. Furthermore, the irradiated tumor and micrometastases were exposed at the same time to the drugs’ cytotoxic effects [32]. The results were in line with previous meta-analysis and several prospective randomized trials [5,8,33–34], in which most of subjects were non-elderly patients.

However, the present study showed no improvements in the D-FFS (75% vs. 73%, *P* = 0.456). A subgroup analysis also showed that chemotherapy could not prevent distant metastases in patients with N2–3 disease (76% vs. 74%, *P* = 0.435). One reason might be that the majority of elderly NPC patients, though they were in excellent physical condition, were administered a fixed lower dose of chemotherapy (DDP 30mg d1–5+ 5-fluorouracil 750mg d1–5) regardless of body size or BSA, possibly mainly worrying about the side effects and toxicity of full-dose cytotoxic chemotherapy. This attenuated-dose regimen possibly decreased the effect of chemotherapy. Another reason might be that two cycles of induction chemotherapy or five cycles of concurrent chemotherapy were still not sufficient to eradicate all the distant micrometastases. Adjuvant chemotherapy may be necessary in order to improve D-FFS rate [5,27,33].

The composite overall response rates between the two groups were similar (99.4% vs. 98.3% for CRT vs. RT, *P* = 0.315). There was a notably higher incidence of toxicity in the CRT group, both hematologic toxicity and nonhematologic toxicity (acute mucositis and weight loss). However, Grade ≥3 haematological toxicity in CRT group remained acceptable (leucopaenia 8.0%, anaemia 6.9%, thrombocytopaenia only in 1.1%). Noteworthy is the fact that 46.0% of patients developed Grade ≥3 mucositis in CRT group. The reason may partially be a function of the use of cisplatin-based regimens, but may also be related to other factors. Advancing age is associated with metabolic changes, higher incidence of comorbidities, and polypharmacia [35–36] which may increase treatment-related toxicities. As Grade ≥3 mucositis increased chemotherapy refusal in elderly NPC patients, the effective supportive care was of critical importance, such as aggressive monitoring, antibiotic therapy for neutropenic fever, and prompt nutritional intervention [32,37].

There are some limitations in our study. Firstly, it is a retrospective study; secondly, the sample was still small because of the strictly matching conditions; at last, all patients were treated with 2D conventional RT, while the widely using of IMRT showed excellent locoregional control in NPC. Therefore, whether combined therapy of IMRT and chemotherapy could bring a survival and adverse effect benefit and the optimal dose and course of chemotherapy for the elderly NPC patients requires further investigation.

## CONCLUSIONS

Based on the above findings, we conclude that standard chemo-radiotherapy can achieve a reasonable local and regional control in elderly NPC patients,with acceptable and reversible acute toxicity. However, distant metastasis remains the dominant failure pattern. When the elderly NPC patients are in a good general condition following a complete evaluation of overall functional status and comorbidity conditions, standard chemo-radiotherapy is worthy of recommendation.

## Supporting Information

S1 Dataset Elderly nasopharyngeal carcinoma database. (XLS)
